# Investigation of the effect of ultrasonography-guided bilateral erector spinae plane block on postoperative opioid consumption and pain scores in patients undergoing hepatectomy: a prospective, randomized, controlled study

**DOI:** 10.1590/1516-3180.2020.0757.R1.08062021

**Published:** 2022-01-17

**Authors:** Gülçin Hacıbeyoğlu, Ahmet Topal, Tevfik Küçükkartallar, Resul Yılmaz, Şule Arıcan, Sema Tuncer Uzun

**Affiliations:** I MD. Assistant Professor, Department of Anesthesiology and Reanimation, Necmettin Erbakan University, Meram Faculty of Medicine, Meram, Konya, Turkey.; II MD. Professor, Department of Anesthesiology and Reanimation, Necmettin Erbakan University, Meram Faculty of Medicine, Meram, Konya, Turkey.; III MD. Professor, Department of General Surgery, Necmettin Erbakan University, Meram Faculty of Medicine, Meram, Konya, Turkey.; IV MD. Assistant Professor, Department of Anesthesiology and Reanimation, Necmettin Erbakan University, Meram Faculty of Medicine, Meram, Konya, Turkey.; V MD. Assistant Professor, Department of Anesthesiology and Reanimation, Necmettin Erbakan University, Meram Faculty of Medicine, Meram, Konya, Turkey.; VI MD. Professor, Department of Anesthesiology and Reanimation, Necmettin Erbakan University, Meram Faculty of Medicine, Meram, Konya, Turkey.

**Keywords:** Hepatectomy, Pain, Analgesia, Ultrasonography, Erector spinae plane block, Opioid consumption, Hepatectomy surgery

## Abstract

**BACKGROUND:**

There is still a debate about what constitutes effective and safe postoperative analgesia in hepatectomy surgery. Erector spinae plane (ESP) block may be an important part of multimodal analgesia application in hepatectomy surgery.

**OBJECTIVES:**

To compare the effects of ultrasound-guided bilateral erector spinae plane block combined with intravenous (iv) patient-controlled analgesia (iv PCA), in comparison with iv PCA alone, in hepatectomy surgery.

**DESIGN AND SETTINGS:**

Randomized prospective single-blinded study in a tertiary university hospital.

**METHODS:**

Fifty patients scheduled for elective hepatectomy surgery were included in the study. Patients were randomized into the ESP group or the control group. In the ESP group, bilateral ESP block was performed preoperatively and iv PCA was used. In the control group, only iv PCA was used. Numerical rating scale (NRS) scores at rest and coughing, analgesic requirements and occurrences of nausea and vomiting were recorded.

**RESULTS:**

Intraoperative and postoperative opioid consumption, rescue analgesia requirement and resting and dynamic NRS scores were significantly lower in the ESP group (P < 0.05). There was no significant difference between two groups in terms of the presence of dynamic pain after the first postoperative hour. While all patients in the control group had nausea and vomiting, 24% of the patients in the ESP group did not have nausea and vomiting.

**CONCLUSION:**

This study showed that ESP block can be used as a part of multimodal analgesia, with the benefit of reducing opioid consumption and postoperative nausea and vomiting in hepatectomy surgery.

**CLINICAL TRIAL REGISTRATION:**

ACTRN12620000466943.

## INTRODUCTION

Hepatectomy is a commonly used treatment option for many benign or malignant liver diseases.^1^Bilateral subcostal incision, surgical retraction and large liver resection, which are all used in hepatectomy surgery, lead to severe postoperative pain in the lower thoracic and abdominal region.

Postoperative analgesia for patients who underwent hepatectomy in protocols for enhanced recovery after surgery (ERAS) is one of the issues which are still discussed and waiting for a solution.^2^The use of intravenous (iv) patient-controlled analgesia (iv PCA) has been demonstrated to be effective in postoperative analgesia, but it should not be ignored that drug metabolism will be influenced in this patient group due to hepatectomy. For this reason, use of multimodal analgesia methods is thought to form a correct approach towards reducing iv opioid consumption.^3^Epidural analgesia provides effective postoperative analgesia following abdominal surgery. However, the changes in coagulation parameters after hepatectomy may pose a risk in patients with epidural catheters.^4^For this reason, safer but easily applicable alternatives are needed for patients who will undergo hepatectomy.

Erector spinae plane (ESP) block is a plane block that was first defined for treating thoracic neuropathic pain and later used for postoperative analgesia in abdominal surgery.^5–7^However, the number of randomized clinical studies indicating the effectiveness of this block in hepatectomy surgery is limited.^8,9^To the best of our knowledge, there are no clinical studies in the literature researching the effectiveness of ESP block in hepatectomies carried out with bilateral subcostal incision. Therefore, we conducted a prospective randomized clinical study, with the prediction that an ESP block at T8 level, in addition to the iv morphine therapy that we apply in our routine practice in hepatectomies carried out with bilateral subcostal incision, would reduce postoperative opioid consumption and pain scores.

## OBJECTIVE

The aim of this study was to compare the effect of ultrasound-guided bilateral erector spinae plane block combined with iv PCA, in comparison with iv PCA alone, in hepatectomy surgery.

## METHODS

### Study design

This study was designed in an academic university hospital as a prospective randomized controlled single-blinded study, in accordance with the principles defined in the Helsinki Declaration. The study was conducted after obtaining approvals from the university's ethics committee (decision number: 2019/243; approval date: November 13, 2019) and from the Ministry of Health Ethics Committee (66175679-514.04.01-E.214738; approval date: December 14, 2019). It was registered in the Australian New Zealand Clinical Trials Register (Trial ID: ACTRN12620000466943). Written informed consent statements were received from all the patients who agreed to participate in the study.

Patients between the ages of 18 and 65 years, presenting with American Society of Anesthesiologists (ASA) physical status I-III, who had been scheduled to undergo elective hepatectomy surgery in which bilateral subcostal incision would be used as the surgical incision, and for whom a self-retaining retractor would be used, were included in the study. Patients were excluded from the study in the following circumstances: obesity (body mass index > 30 kg/m^2^); local skin infection in the area where the needle would be inserted; known allergy to any of the drugs to be used in the study; coagulopathy; chronic opioid consumption; inability to use the PCA device; advanced liver failure; kidney failure; or lack of agreement to participate in the study.

### Patient groups and randomization

The patients were randomized and grouped by using the sealed opaque envelope technique. A researcher who was not included in the study performed this procedure. The patients who would not have the block and would only use an iv PCA device for postoperative analgesia comprised the control group. The patients who would undergo preoperative bilateral ESP block and use an iv PCA device postoperatively comprised the ESP group.

### Anesthesia application

The same general anesthesia method was applied to all the patients, and the hepatectomy operation was carried out by the same surgical team. Before the operation, all the patients were told about numerical rating scale (NRS) scores for assessing their postoperative pain severity and how to use the iv PCA device. The patients’ demographic data and ASA scores were recorded. Routine monitoring and neuromuscular transducer (NMT) monitoring (SJC17200038HA, GE Healthcare, Helsinki, Finland) were performed in the operating room.

Among the patients sedated with 0.03 mg/kg of midazolam (Midolam, Mefar, Istanbul, Turkey), those in the ESP group were subjected to bilateral ESP block with ultrasonography before the induction of anesthesia. In all patients, anesthesia was induced with 40 mg of lidocaine (Jetmonal, Adeka, Samsun, Turkey), 2 mg/kg of propofol (Propofol 1% Fresenius, Fresenius Kabi AB, Uppsala, Sweden), 1 μg/kg of remifentanil (Rentanil, VEM, Tekirdağ, Turkey) and 0.6 mg/kg of rocuronium (Myocron, VEM, Tekirdağ, Turkey). The anesthesia was maintained through inhalation of 0.5-1 MAC desflurane (Suprane, Baxter Healthcare, Puerto Rico, United States) and infusion of remifentanil. The analgesic requirement was monitored using the surgical pleth index (SPI) (SJB17230028HA, GE Healthcare, Helsinki, Finland); the remifentanil infusion dose was set to a SPI below 50; and the total intraoperative remifentanil consumption was recorded.

The patients underwent invasive artery monitoring and right internal jugular vein catheterization via ultrasonography. The surgery was carried out by making a bilateral subcostal incision and using a self-retaining retractor. The duration of the surgery and the surgery performed (right hepatectomy or left hepatectomy) were recorded.

Thirty minutes before the end of the operation, 0.1 mg/kg of iv morphine (Morphine HCl, Idol, Istanbul, Turkey) was administered for postoperative analgesia. The antiemetic ondansetron (Zofran, GlaxoSmithKline, Research Triangle Park, England) was administered to patients at a dose of 0.1 mg/kg, iv. Patients whose muscle relaxation was reversed with sugammadex (Bridion, Patheon Manufacturing Services, North Carolina, United States) were extubated when their Train of Four (TOF) values were ≥ 90%, and they were taken to the post-anesthesia care unit (PACU). Here, an iv PCA device (BodyGuard 575 Pain Manager, Caesarea Medical Electronics GmbH, Lichtenstein, Germany) was attached to patients for postoperative analgesia. PCA was programmed as 1 mg/ml of morphine without a basal infusion dose, as 1 ml per bolus, with a lock-out time of six minutes. Patients were followed up until their modified Aldrete score reached 9 in the PACU and were then transferred to the intensive care unit of the related clinic.

### ESP block application

All the blocks were carried out about 30 minutes before induction of anesthesia. This was done by researchers who would not monitor the postoperative data from the patients (GH, AT). The patients were placed in the prone position, and the skin was prepared with 10% povidone iodine (Poviderm, Necm Chemistry, Istanbul, Turkey). The position of the T7 vertebra at the level of the lower ends of the scapula was determined, and the T8 vertebra one level below this was then detected by palpation.

The T8 spinous process was first seen in the horizontal plane on the midline by using a linear probe covered with a sterile cover at 8 mHz frequency, by means of ultrasonography (Esaote MyLab Six CrystaLine, Genova, Italy). The probe was then turned to the longitudinal plane, and the transverse process was seen approximately 3 cm from the midline to the left lateral and the erector spinae muscle was seen on it.

A 22-gauge 80-mm block needle (Sonoplex, Pajunk Medical, Geisingen, Germany) was advanced craniocaudally in-plane, and the transverse process was touched. The needle was then minimally retracted, and its positioning between the erector spinae muscle and the transverse process was confirmed through hydro-dissection. Following this, 20 ml of 0.375% bupivacaine hydrochloride (Buvicaine, Polifarma, Tekirdağ, Turkey) + 4 mg of dexamethasone (Dekort, Deva, Tekirdağ, Turkey) were injected, and simultaneous local anesthetic dispersion was monitored by means of ultrasonography. The same procedure was performed on the right side.

Loss of hot-cold sensation below and above the bilateral T8 dermatome level, 20 minutes after the block had been performed, was considered to represent successful blocking. The blocked dermatome levels were recorded.

### Pain assessment and analgesia protocol

Postoperative pain scores and analgesic requirements were assessed by a research assistant who was blinded to the groups, in the PACU and surgical service. The pain severity was assessed both at rest and during coughing. The NRS during coughing was evaluated as dynamic NRS (DNRS), and if there was a difference of two points or more in relation to the resting NRS, this was defined as the presence of dynamic pain. The resting NRS and DNRS scores at the postoperative 10^th^minute, 1^st^hour, 6^th^hour, 12^th^hour and 24^th^hour and morphine consumption at the 1^st^hour, 6^th^hour, 12^th^hour and 24^th^hour were recorded. Rescue analgesia was applied according to the resting NRS scores. If NRS > 4, this was considered to represent inadequate analgesia, and 0.5 mg/kg of iv meperidine (Petisel, Haver, İstanbul, Turkey) was administered. After 30 minutes, the patient was re-evaluated and, if NRS was still > 4, 0.5 mg/kg of iv meperidine was added. Rescue analgesic requirement and nausea-vomiting over the first postoperative 24 hours were recorded. The severity of nausea was evaluated by the patients on a four-point scale (0: none; 1: mild; 2: moderate; or 3: severe). In the presence of moderate or severe nausea-vomiting, patients were administered additional ondansetron at a dose of 0.1 mg/kg iv.

### Primary and secondary outcome criteria

The primary outcome criterion of the study was total morphine consumption over the first postoperative 24 hours. The secondary outcome criteria were the resting and dynamic NRS scores at five different time points (postoperative 10^th^minute, 1^st^hour, 6^th^hour, 12^th^hour and 24^th^hour), intraoperative remifentanil consumption and total rescue analgesic requirement over the first postoperative 24 hours. Besides these measurements, changes in the presence of dynamic pain, postoperative nausea-vomiting, dermatome levels in patients who underwent the block, duration of surgery and surgery applied (right hepatectomy or left hepatectomy) were also evaluated.

### Sample size calculation

The sample size for this study was calculated using the G*Power software, version 3.1.9.4 for Windows (Universität Düsseldorf, Düsseldorf, Germany), based on a pilot study with 10 patients in each group. Occurrence of a reduction in morphine consumption of at least 30% over the first postoperative 24 hours in the ESP group, compared with the control group, was accepted as clinically significant. According to the pilot study results, morphine consumption over the first postoperative 24 hours was 61.4 mg ± 14.7 mg (mean ± standard deviation, SD) in the control group, while it was 39.2 mg ± 14.7 mg in the ESP group. The sample size required for both groups, with 90% power and an error of 0.01 (two-tailed), was calculated as 21 patients. Considering possible patient dropouts, it was planned to include 25 patients in each group (50 patients in total).

### Statistical analysis

The data obtained in this study were evaluated using the Statistical Package for the Social Sciences (SPSS) software, version 23.0 (IBM SPSS 23.0 for Windows, Armonk, New York, United States). The frequencies of general demographic characteristics and the descriptive statistical values of all time-dependent measurements were specified. The Shapiro-Wilk test was applied if n < 30, and the Kolmogorov-Smirnov test if n > 30, during examination of the normality of the scores between the groups. If P < 0.05, the values were considered not to have normal distribution between the groups, and if P > 0.05, the values were assumed to have a normal distribution between the groups. After the normality test, the Mann-Whitney U test was applied to investigate differences between the groups. The chi-square test was performed to investigate intergroup dependence in the categorical data. While examining differences and dependence between the groups, 0.05 was used as the significance level. If P < 0.05, it was accepted that there was a significant difference between the groups. The Wilcoxon signed rank test was used to examine differences between intragroup time-dependent measurement values. If P < 0.05, the measurement values were considered to differ according to time.

## RESULTS

**Figure 1:**
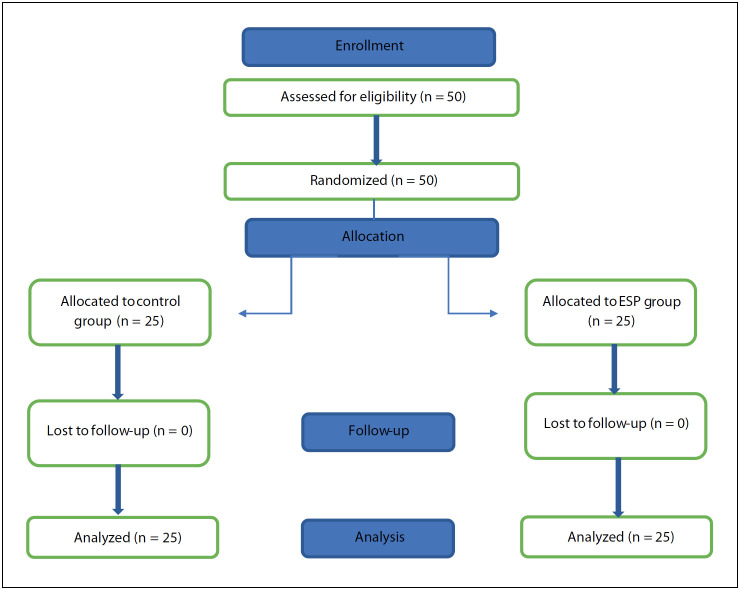
Flowchart of the study.

A total of 50 patients were included in the study, and no patients were excluded from the study (Figure 1). The demographic and surgical data of the two groups were similar to each other (Table 1).

**Table 1:** Assessment of demographic and operative data (mean ± standard deviation)

	Control group (n = 25)	ESP group (n = 25)	P-value
Sex (male/female)	19/6	19/6	1.000
Age (year)	47.6 ± 14.6	44.6 ± 17.1	0.484
Weight (kg)	78.7 ± 5.4	76.8 ± 7.0	0.157
Height (cm)	172.5 ± 6.8	172.9 ± 9.5	0.838
BMI (kg/m^2^)	26.6 ± 1.9	25.8 ± 2.5	0.240
ASA (I/II/III)	2/18/5	7/12/6	0.394
Duration of surgery (min)	234.4 ± 43	216.2 ± 38.2	0.06
Type of surgery (R/L)	18/7	18/7	1.000

ESP = erector spinae plane; BMI = body mass index; ASA = American Society of Anesthesiologists; type of surgery: R = right hepatectomy, L = left hepatectomy.

Intraoperative remifentanil consumption was significantly lower in the ESP group (P < 0.01). Similarly, morphine consumption and rescue analgesic (meperidine) requirement over the first postoperative 24 hours were much lower in the ESP group (P < 0.01) (Table 2).

**Table 2:** Assessment of intraoperative and postoperative analgesia requirements

	Control group (n = 25)	ESP group (n = 25)	P-value
Intraoperative remifentanil (mg)	4.6 ± 1.1	3.2 ± 0.9	**0.000**
Postoperative morphine (mg)	96.3 ± 38.7	49.7 ± 16.9	**0.000**
Postoperative meperidine (mg)	109.0 ± 30.37	55.6 ± 39.74	**0.000**

ESP = erector spinae plane.

**Figure 2:**
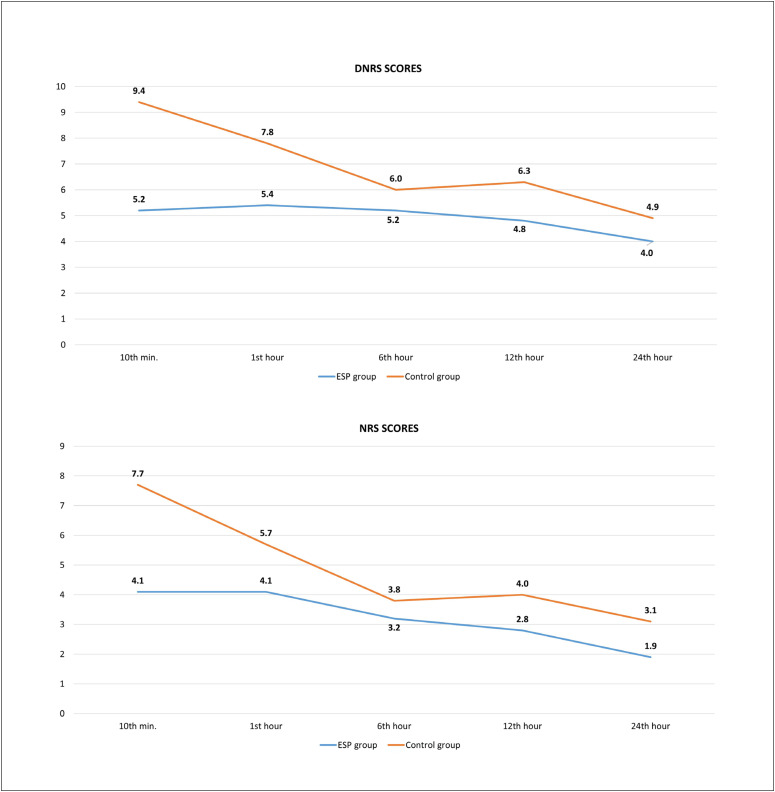
Changes in numerical rating scale (NRS) and dynamic numerical rating scale (DNRS) scores between groups over time.

Postoperative NRS scores were significantly lower in the ESP group (P = 0.000, P = 0.000, P = 0.019, P = 0.000 and P = 0.000, respectively) at all time points (10^th^minute, 1^st^hour, 6^th^hour, 12^th^hour and 24^th^hour). Likewise, DNRS scores were also significantly lower in the ESP group at all time points (P = 0.000, P = 0.000, P = 0.018, P = 0.000 and P = 0.020, respectively) (Figure 2).

Considering the presence of dynamic pain, this was observed in all the patients in the control group at the 10^th^minute, while it was only observed in 16% of the patients in the ESP group (P = 0.000). While dynamic pain was present in 88% of the control group at the 1^st^hour, it was present in 24% of the ESP group (P = 0.000). There was no significant difference in the presence of dynamic pain at the other times evaluated (Table 3).

**Table 3:** Variation in the presence of dynamic pain between the groups over time

Presence of dynamic pain	Group				P-value
	Control group		ESP group		
	n	%	n	%	
10^th^minute	25	100.0	4	16.0	**0.000**
1^st^hour	22	88.0	6	24.0	**0.000**
6^th^hour	23	92.0	22	88.0	1.000
12^th^hour	24	96.0	23	92.0	1.000
24^th^hour	16	64.0	22	88.0	0.098

ESP = erector spinae plane.

**Figure 3:**
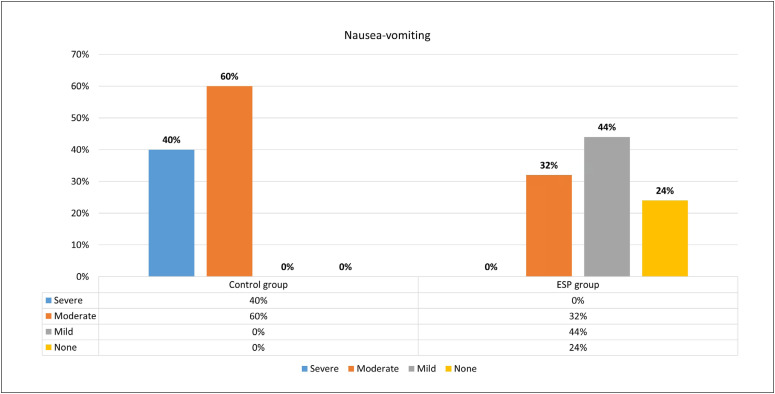
Postoperative presence of nausea-vomiting.

Evaluation of postoperative nausea-vomiting showed that this was present in all patients in the control group, and it was severe in 40%. On the other hand, 24% of the patients in the ESP group did not have nausea-vomiting (Figure 3). It was discovered that an average of 7.36 ± 0.9 dermatome levels (minimum 6, maximum 9) were blocked in patients who had ESP block (Table 4).

**Table 4:** Dermatome levels blocked among the patients

Patient	Dermatome level	Patient	Dermatome level
Patient 1	T4-T10 **(7)**	Patient 14	T4-T12 **(9)**
Patient 2	T4-T12 **(9)**	Patient 15	T5-T11 **(7)**
Patient 3	T4-T10 **(7)**	Patient 16	T5-T11 **(7)**
Patient 4	T4-T10 **(7)**	Patient 17	T5-T12 **(8)**
Patient 5	T5-T12 **(8)**	Patient 18	T5-T11 **(7)**
Patient 6	T5-T12 **(8)**	Patient 19	T6-T12 **(7)**
Patient 7	T5-T12 **(8)**	Patient 20	T6-T11 **(6)**
Patient 8	T4-T9 **(6)**	Patient 21	T6-T12 **(7)**
Patient 9	T4-T12 **(9)**	Patient 22	T6-T12 **(7)**
Patient 10	T4-T10 **(7)**	Patient 23	T5-T12 **(8)**
Patient 11	T4-T10 **(7)**	Patient 24	T5-T12 **(8)**
Patient 12	T5-T10 **(6)**	Patient 25	T5-T11 **(7)**
Patient 13	T5-T11 **(7)**		

T = thoracic.

## DISCUSSION

There is still a debate about what constitutes effective and safe postoperative analgesia in hepatectomy surgery. In open-technique hepatectomies, postoperative pain arises from the surgical incision or diaphragmatic irritation, or it is of visceral origin.^10^In ERAS protocols, regional effective methods have been recommended for analgesia, postoperative mobilization and recovery after hepatectomy.^2^Use of iv opioids seems to be the most important part of multimodal analgesia in hepatectomy surgery.^3^However, there is an increasing tendency towards reducing morphine consumption, due to its toxic, immunological and oncogenic effects on the liver, although this is a controversial movement.^11^Nonetheless, additional methods need to be used to reduce the side effects of opioids in postoperative pain management.^12^

Although debate continues regarding the visceral effectiveness of the block, we predicted that bilateral ESP block from the lower thoracic region could contribute to postoperative analgesia in hepatectomy surgery, as observed in the cadaver studies.^5,13,14^We applied the block before making the surgical incision since this would reduce the effect of neuromodulation and improve postoperative pain control.^15^Considering that the mean duration of surgery was 216.2 ± 38.2 minutes in the ESP block group, we assume that the main effect of the block was on intraoperative remifentanil consumption. Thus, while the consumption of remifentanil was 3.2 ± 0.9 mg in patients who had ESP block, it was 4.6 ± 1.1 mg in the control group. In the postoperative period, it was found that NRS and DNRS scores and morphine and rescue analgesic consumption were significantly lower in the ESP block group, in all timeframes. We attribute this result both to the reduction of postoperative hyperalgesia through decreased intraoperative remifentanil consumption and to the analgesic effectiveness of the block.

Different results have been obtained in the literature with regard to ESP block applied in different volumes and concentrations in different types of abdominal surgery. Tulgar et al.^16^evaluated oblique subcostal transversus abdominis plane block and ESP block among laparoscopic cholecystectomy patients, and showed that resting and dynamic NRS scores in both blocks significantly decreased in the first three hours, compared with the control group, which had no block application. After the third hour, no difference in dynamic NRS scores was found. In their study, the ESP block was carried out at T9 level with 20 ml of 0.375% bupivacaine, but dexamethasone was not used.

In a study evaluating 182 patients who underwent ESP block, cases with suspected local anesthetic toxicity were reported.^17^This block, which has a similar effect to paravertebral and intercostal blocks, may have unforeseen systemic toxic effects. This situation requires greater care regarding drug dose and volume adjustment, particularly in bilateral blocks. In our study, we carried out our bilateral block application with 20 ml of 0.375% bupivacaine + 4 mg of dexamethasone. We did not find any signs of systemic toxicity in any of the 25 patients on whom we performed the block. We think that the drug concentration, volume and use of dexamethasone were effective in relation to block efficiency.

Steroid injections may potentially contribute to analgesia by suppressing abnormal pain transmission in damaged nerves, providing the modulation of transmission in normal nerves, and showing an anti-inflammatory effect.^18^There has only been limited use of adjuvant in ESP block in clinical studies in the literature that were conducted to assess postoperative pain.^8,19^In our study, the NRS and DNRS scores, which were high in the first hours in the ESP group, were found to gradually decrease over other timeframes. Although we did not have a control group without dexamethasone for evidence, we think that the emergence of the anti-inflammatory properties of dexamethasone and the rescue analgesics used had an effect on this result. For this reason, we think that different randomized studies should be conducted to support our study, in order to specify the effect of dexamethasone. Furthermore, we investigated the effect of the block in the first postoperative 24 hours. We are of the opinion that it should be investigated whether adjuvant-enhanced ESP block without catheter insertion has an effect on postoperative pain for more than 24 hours.

In the literature, there are two randomized studies investigating the effectiveness of ESP block in hepatectomy surgery.^8,9^In both studies, ESP block was found to reduce the intraoperative remifentanil consumption. Thus, we think that these results provide confirmation of the visceral analgesic property of the block. Kang et al.^9^used an ESP block at T8 bilaterally with 20 ml of 0.5% ropivacaine and compared this with use of 400 mcg of intrathecal morphine, in live liver donors who underwent laparoscopic hepatectomy. The pain scores in patients who underwent ESP block were higher than in those who received intrathecal morphine, but they were within acceptable limits, and postoperative nausea was found to be similar to what we saw in our study. In the study of Kang et al.,^9^multimodal analgesia was administered using a large number of agents, and long-term follow-up such as after 72 hours postoperatively was performed. The pain score in the first 24 hours was 1.3 in the intrathecal morphine group, while it was 2.5 in the ESP group. In our study, the resting NRS score was determined as 1.9 for the ESP group at the 24^th^hour, whereas the DNRS score was found to be 4.0 for the ESP group. We attribute our higher dynamic pain score to the open technique that was applied for the surgery, the use of retractors and the limited range of analgesics that we used, especially our use of non-steroidal anti-inflammatory drugs (NSAIDs).

Our aim in performing the block at T8 was to relieve the patients’ painful breathing, caused by upper abdominal pain that resulted from retraction, and thus to reduce both the dynamic and resting pain scores, which was also successful. However, the difference in the presence of dynamic pain was found to disappear after the first hour. Additionally, although the NRS scores in the ESP group were lower than those in the control group, an average score of 4.1 was detected in the first hours.

Considering the duration of surgery, the postoperative analgesic property of the block may have been reduced, and this may have had a negative effect on resting and dynamic pain scores and on opioid consumption. To prevent this situation, performing the block at more than one level or using a catheter may be preferred options. However, this may be more invasive and open to complications, compared with a single-level injection. For this reason, performing the block with different volumes and concentrations and using different adjuvant agents (such as dexmedetomidine) can be regarded as aims for future studies.

Another reason for this clinical picture may be that the block was not supported with multimodal analgesic drugs. For instance, if the block had been supported through use of NSAIDs or paracetamol, the duration of the positive effect on the presence of dynamic pain could have been extended. We believe that more comprehensive studies should be conducted on this subject.

In the meta-analysis by Kendall et al.,^20^it was reported that one of the most significant effects of ESP block was on postoperative nausea-vomiting, and that this resulted from both the decrease in opioid consumption and the high analgesic efficiency of the block. In our study, postoperative opioid consumption was observed to be significantly lower in the ESP block group. While no nausea-vomiting was encountered in 24% of the patients who underwent the block, 44% had mild and 32% moderate nausea and vomiting. In the control group, on the other hand, nausea and vomiting were observed, and 40% of the cases were severe. We think that we significantly reduced postoperative nausea-vomiting through decreasing morphine consumption over the first 24 hours and through increasing analgesic efficacy with the ESP block.

Our study had some limitations. The methodology was planned in a single-blind manner, which may have affected the objectivity of the results. Another factor with a possible effect on the outcome criteria was that patient homogenization could not be achieved. To fully explain the true analgesic effectiveness of the ESP block, we formed the control group from patients who received systemic analgesics alone. However, lack of a block group without dexamethasone may have prevented us from evaluating the net effect of the adjuvant drug. In addition, dexamethasone, which was used as an adjuvant in the block, had a systemic analgesic effect as well as prolonging the duration of the block. Non-administration of intravenous dexamethasone to the control group may have affected the objective evaluation of analgesic efficacy between the groups. Another limitation was that the data were limited to a short period of time, for such a major surgery. We did not have a chance to determine the duration of the positive effect of the block that we performed. In this study, we administered opioid alone over the first 24 hours, while avoiding NSAIDs and paracetamol because of their hepatotoxic effects, considering that they might have negative effects on the bleeding profile. This can be considered to be another limitation. Much lower NRS and DNRS scores could have been obtained through using analgesics with different effect mechanisms. We preferred to use a concentrated rate of 0.375% in the bilateral block. Our preoperative application of the block may have caused the signs of local anesthesia toxicity to be masked through general anesthesia. This may have prevented us from objectively assessing the reliability of the bilaterally applied block at this concentration.

## CONCLUSION

Ultrasound-guided bilateral erector spinae plane block significantly reduced intraoperative and postoperative opioid use in hepatectomy surgery carried out by means of a bilateral subcostal incision and also relieved postoperative pain experienced during coughing. It also significantly reduced postoperative nausea and vomiting. Erector spinae plane block may be an important part of multimodal analgesia application in hepatectomy surgery, in terms of its easy application and safety, provision of effective analgesia and reduction of opioid side effects. On the other hand, there is a need for multicenter randomized studies aimed at prolonging and strengthening the effect of the block, such as through an appropriate drug dosage and concentration, and through catheter use.
